# Emergence of nosocomial associated opportunistic pathogens in the gut microbiome after antibiotic treatment

**DOI:** 10.1186/s13756-021-00903-0

**Published:** 2021-02-15

**Authors:** Isaac Raplee, Lacey Walker, Lei Xu, Anil Surathu, Ashok Chockalingam, Sharron Stewart, Xiaomei Han, Rodney Rouse, Zhihua Li

**Affiliations:** Division of Applied Regulatory Science, Office of Clinical Pharmacology, Office of Translational Sciences, Center for Drug Evaluation and Research, Food and Drug Administration, Silver Spring, MD USA

**Keywords:** Antibiotic resistance, Metagenomics, Nosocomial, Microbiota, Antibiotic therapy

## Abstract

**Introduction:**

According to the Centers for Disease Control’s 2015 Hospital Acquired Infection Hospital Prevalence Survey, 1 in 31 hospital patients was infected with at least one nosocomial pathogen while being treated for unrelated issues. Many studies associate antibiotic administration with nosocomial infection occurrence. However, to our knowledge, there is little to no direct evidence of antibiotic administration selecting for nosocomial opportunistic pathogens.

**Aim:**

This study aims to confirm gut microbiota shifts in an animal model of antibiotic treatment to determine whether antibiotic use favors pathogenic bacteria.

**Methodology:**

We utilized next-generation sequencing and in-house metagenomic assembly and taxonomic assignment pipelines on the fecal microbiota of a urinary tract infection mouse model with and without antibiotic treatment.

**Results:**

Antibiotic therapy decreased the number of detectable species of bacteria by at least 20-fold. Furthermore, the gut microbiota of antibiotic treated mice had a significant increase of opportunistic pathogens that have been implicated in nosocomial infections, like *Acinetobacter calcoaceticus/baumannii* complex, *Chlamydia abortus*, *Bacteroides fragilis*, and *Bacteroides thetaiotaomicron*. Moreover, antibiotic treatment selected for antibiotic resistant gene enriched subpopulations for many of these opportunistic pathogens.

**Conclusions:**

Oral antibiotic therapy may select for common opportunistic pathogens responsible for nosocomial infections. In this study opportunistic pathogens present after antibiotic therapy harbored more antibiotic resistant genes than populations of opportunistic pathogens before treatment. Our results demonstrate the effects of antibiotic therapy on induced dysbiosis and expansion of opportunistic pathogen populations and antibiotic resistant subpopulations of those pathogens. Follow-up studies with larger samples sizes and potentially controlled clinical investigations should be performed to confirm our findings.

## Introduction

Patients who acquire a nosocomial infection have approximately a 1 in 10 chance of mortality during their hospitalization [1]. In 2009, hospital acquired infections (HAIs) were estimated to cost about 45 billion dollars in direct costs [2]. One of the most common primary nosocomial infections is urinary tract infection (UTI). Recent research suggests oral antibiotic treatment of a primary nosocomial infection, like a UTI, is indirectly linked to secondary nosocomial antibiotic resistant infections [3, 4]. Secondary nosocomial infections are often multi-drug resistant opportunistic pathogens, such as, the *Acinetobacter calcoaceticus/baumannii* complex or *Clostridioides difficile *[5–7]. The human gut and intestinal microbiome are heavily populated with diverse microbial organisms. It is well known that antibiotic treatment reduces the diversity and number of bacteria in the digestive microbiome. Furthermore, it is well established that normal microbiota helps prevent the growth of pathogenic bacteria [8, 9]. We acknowledge the great amount of work showcasing antimicrobial therapy induced *Clostridiodes difficile* infection [10]. However, to our knowledge there is very little other direct experimental evidence that antibiotic treatment specifically selects for opportunistic pathogens associated with HAI in the gut microbiome.

The introduction of high-throughput sequencing technology has led to powerful advances in microbial community ecology composition and function assessments. Despite the extensive advancements in high-throughput sequencing there is a large portion of the human gut microbiome composition still uncharacterized. The two major sequencing methods used to assess microbial communities of human systems are amplicon sequencing and whole metagenome sequencing (WMS). While amplicon sequencing may provide enough information to assess composition, there are biases present, from amplification and primer selection, that cannot be ignored [11]. Additionally, WMS is the preferred method for strain assessment and functional profiling of diverse microbiomes. Traditionally, taxonomy profiling of WMS data relies on mapping each individual read to reference databases. However, a common issue with such a method is the large portion of unmappable reads from uncharacterized species and subspecies present in the microbiome. Most reference databases are built from cultivated microbes. Unfortunately, many species present in microbial ecosystems are not able to be cultivated and therefore reference genome databases only represent a portion of species present. Even culture-independent genomic approaches have a large presence of unexplored microbial populations [12, 13].

**Fig. 1:**
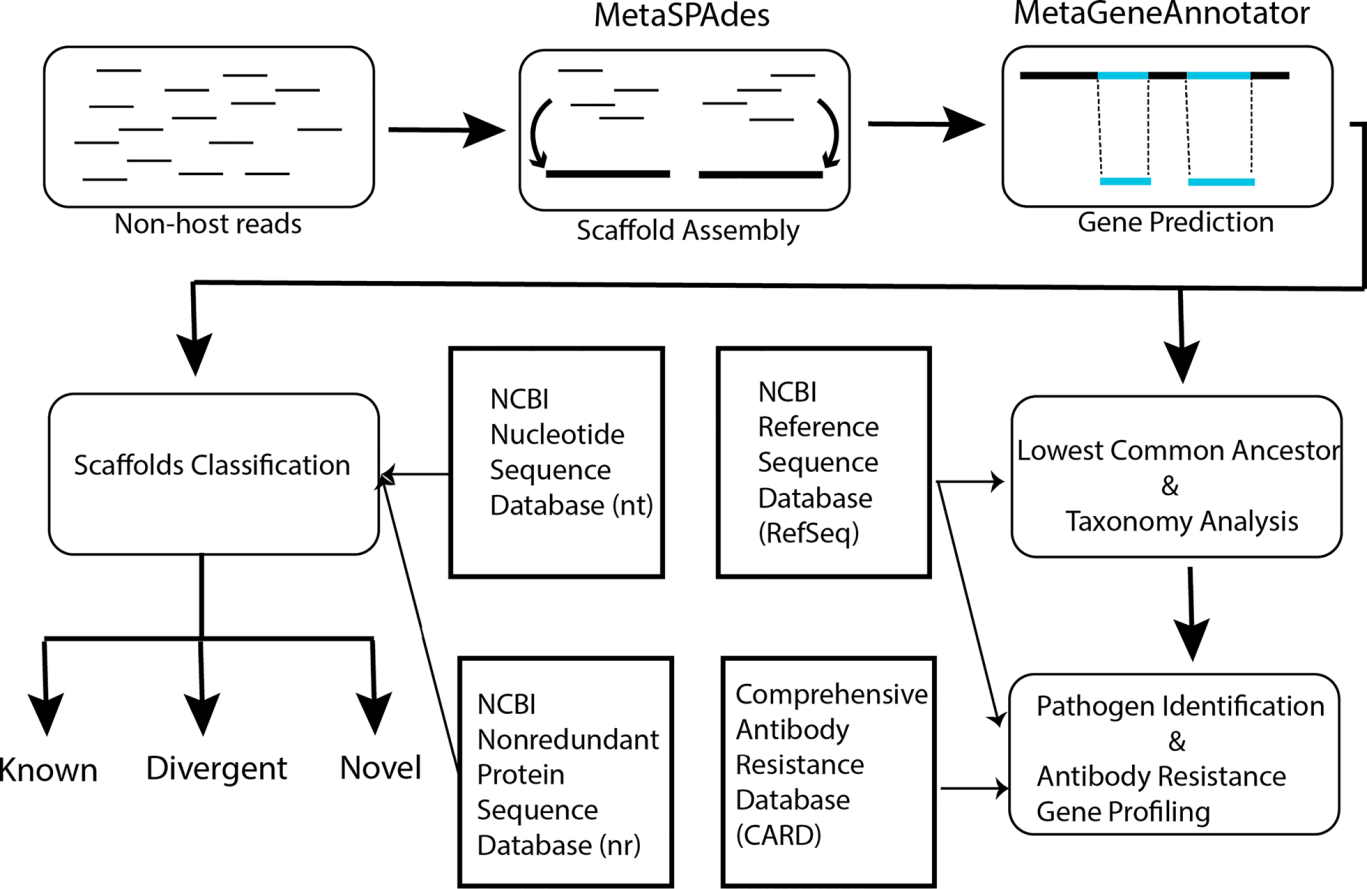
Bioinformatics pipeline. While traditional metagenome analysis methods align reads with sequences in reference databases and perform taxonomy assignment directly, the method we used assembles reads into longer scaffolds before mapping to reference databases, and relies on lowest common ancestor (LCA) analysis to perform taxonomy assignment because scaffolds can potentially harbor multiple predicted genes. The longer length of scaffolds also makes it possible to classify scaffolds into one of the known, divergent, or novel categories based on their overall sequence similarity with reference databases. Publicly available tools used are annotated on top of boxes where used in the pipeline. Boxes in the center refer to publicly available databases used in the pipeline. NCBI: National Center for Biotechnology Information

Recently we applied a traditional read-mapping based method to the metagenome analysis of antibiotic treated mice and revealed a decrease of gut microbiota diversity and enrichment of antibiotic resistance genes (ARGs) in the gut [14]. However, as with many other studies, the read-mapping method left a large portion of the reads uncharacterized. In this paper we implemented an inhouse pipeline that incorporates metagenome assembly, gene prediction, and lowest common ancestor taxonomic assignment (Fig. 1). Such a method not only greatly increased the portion of characterizable reads, but also expanded upon the direct evidence that antibiotic treatment for a common healthcare-associated infection selects for a gut antibiotic resistant reservoir of opportunistic pathogens associated with HAIs, in addition to the well-established digestive microbiome dysbiosis.

## Materials and methods

### Animal studies

Experiments were conducted on BALB/c female mice (Taconic Biosciences, Derwood, MD) 8 – 10 weeks old. The same strain, sex and age mice were obtained from the same vendor and the same location in the vendor facility. Mice were randomly allocated to control or treatment groups (Ampicillin, Ciprofloxacin, or Fosfomycin treated groups), each with 4 animals. Not all animals yielded sufficient amount of sequencing reads (> ~ 10 million per sample) and in the end 3 animals per group were used for subsequent sequencing analysis. To recapitulate the most common nosocomial infection, a urinary tract infection model previously described [15–17] was used. All mice, except two of the three control mice, were inoculated with CFT073 uropathogenic *E.coli* (ATCC, Manassas, VA) to create the UTI model. Our previous study showed no discernable difference in the gut microbiome of naïve and urinary tract infection model mice [14]. Treatment groups include antibiotics Ampicillin, Ciprofloxacin, and Fosfomycin. Ampicillin treatment was with ampicillin trihydrate (Sigma, St. Louis, MO) 200 mg/kg in 0.1 M HCl twice daily with an 8-h interval for three consecutive days. Ciprofloxacin treatment was with a 5% oral suspension 50 mg/kg (Bayer HeathCare, Whippany, NJ) twice daily with an 8-h interval for three consecutive days. Fosfomycin treatment was with Monurol® Fosfomycin tromethamine 1000 mg/kg (Forest Pharmaceutical Inc., St Louis, MO) once daily for three consecutive days. Fecal samples to be sequenced were collected from the distal ileum and proximal colon after the third day of treatment. All animal studies were done in accordance with an approved protocol from the Institutional Animal Care and Use Committee of the White Oak Federal Research Center.

### DNA extraction and sequencing

Genomic DNA extraction and sequencing was completed as previously described [14]. Briefly, collected fecal samples had DNA extracted using QIAamp DNA stool mini kit (Qiagen, Germantown, MD) with slight modifications to the protocol. Libraries were prepared with the Nextera DNA Library Prep Kit (Illumina, San Diego, CA) and sequenced on the NextSeq 500 Sequencing system (Illumina, San Diego, CA).

### Host reads removal and metagenomic assembly

Sample outputs were processed into raw read fastq files with bcl2fastq2 (version 2.18.0.12, Illumina Inc.). Host reads were removed by first aligning reads to an indexed mouse genome (generated by downloading all sequences from the National Center for Biotechnology Information (NCBI) databases with the taxonomy ID of 10,090) using BWA-MEM (version 0.7.12), then unmapped reads were extracted using SAMtools (version 0.1.18) with parameters “-h -f 4”, and the output was converted to fastq with Picard-Tools (version 2.1.1) SamToFastq function. Assembly was performed with SPAdes (version 3.12.0) using the recommended parameters “-k 21,33,55,77” with the –meta flag to initiate MetaSPAdes mode for paired end samples (Control, Ampicillin and Ciprofloxacin cohorts) [18] and -s flag for single end samples (Fosfomycin) [19]. All scaffolds with greater than 500 bps were retained for downstream analysis. To further select for microorganism scaffolds another round of host read removal was performed.

### Gene prediction and scaffold divergence classification

The MetaGeneAnnotator program [20] was used to predict genes within retained scaffolds for each sample. The resulting text file output was converted to BED format using an inhouse python script (https://github.com/FDA/metagenome). Gene sequences were extracted from the retained scaffold using BEDTools [21] and the BED file of results, generating a gene prediction results fasta file. We characterized each scaffold using methods described by Kowarsky et al. [22]. Briefly, scaffolds were characterized as either novel, divergent, or known. Novel scaffolds were all those with BLASTn alignments that spanned less than 20% of bases and had an average gene identity below 60%. Scaffolds were assigned to the known category if their average gene identity was greater than 80%. Divergent scaffolds were all those that neither fit into novel or known. To determine gene identity of each scaffold each samples’ predicted genes results fasta file was aligned with BLASTx to the NCBI nonredundant protein database (https://ftp.ncbi.nlm.nih.gov/blast/db/FASTA/nr.gz). To determine alignment coverage scaffolds greater than 500bps in length were aligned with BLASTn to NCBI nucleotide database (https://ftp.ncbi.nlm.nih.gov/blast/db/FASTA/nt.gz). BLASTn alignments to determine coverage were completed with the following parameters “-task megablast -evalue 1e-6 -max_target_seqs 1 -best_hit_score_edge 0.05 -best_hit_overhang 0.05 -window_size 0 -percent_identity 90”. BLASTx alignments to determine percent identity were completed with the following parameters “-max_target_seqs 1 -evalue 1e-6”. All BLAST alignments were completed using a local installation of the BLAST command line application BLAST + (version 2.3.0) [23].

### Taxonomy, opportunistic pathogen, and antibiotic resistance profiling

Taxonomic assignment for scaffolds was performed in three steps. First, the top 10 hits from each predicted gene in a BLASTx alignment to the NCBI RefSeq database sorted by bitscore were retained and stored in an in-house developed SQLite database through the R package RSQLite (https://cran.r-project.org/package=RSQLite). The top scoring hits for a predicted gene with at least 60% positive scoring matches were retained for lowest common ancestor analysis. Second, the full length of each scaffold was mapped to the NCBI NT database through BLASTn, and any scaffold with over 90% percent identity with any mouse sequences were removed. Third, the taxon id of the lowest common ancestor of all the retained top BLASTx hits was assigned to the scaffold. To determine the abundance of opportunistic pathogens we counted how many reads of each sample aligned to the genome of opportunistic pathogens using Bowtie2 (version 2.1.0). For plotting purpose, normalization was performed by multiplying mapped read counts by 1 million and then dividing by the total number of reads from the respective sample (reads per million). To determine the profile of antibiotic resistance genes for specific species present we aligned predicted genes using BLASTx to the CARD database (August, 2019) [24]. Counts were tallied for every read that was aligned to the CARD database and was associated with a scaffold from an opportunistic pathogen.

### Statistical analysis

ARG counts and counts of reads mapping to opportunistic pathogens were normalized using edgeR’s default normalization method (TMM normalization) and edgeR’s analysis functions, glmFit, and glmLRT were used to estimate the dispersion and test for differential abundance [25].

## Results

### Scaffold assembly increased percent of reads mapped

**Fig. 2:**
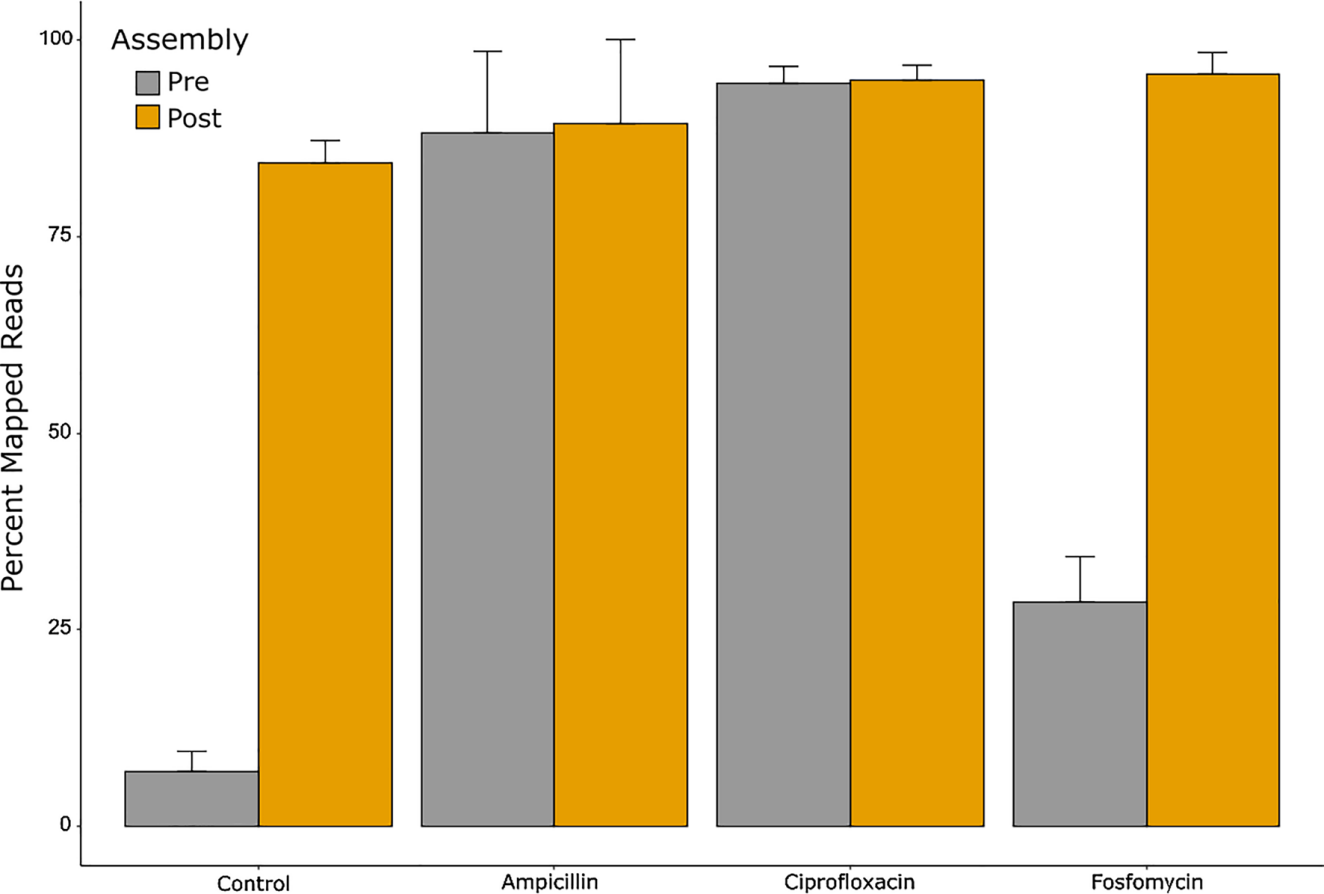
Comparison of Mapped Reads before and after assembly. Pre-assembly mapped reads (Grey): percentage of reads that can be mapped to the NCBI nucleotide database. Post-assembly mapped reads (Gold): percentage of read that can either be mapped to the NCBI nucleotide database or be assembled into a gene that can be mapped to the NCBI nucleotide or protein databases. Control, Ampicillin, and Ciprofloxacin cohorts had scaffolds assembled using pair-end reads. Fosfomycin cohort contigs were assembled using single end reads. See Methods for details

To characterize the microbiome, filtered reads were mapped using BLASTn to the NCBI nucleotide database. To increase mapping rates, we performed de novo assembly of reads into scaffolds. A total of 313,519 scaffolds greater than 500 bps were created for the Control cohort. Ampicillin, Ciprofloxacin, and Fosfomycin-treated groups had 12,438, 25,171, and 5,331 scaffolds greater than 500 bps generated, respectively. Assembly in conjunction with de novo gene prediction (see Methods) increased the average percent of reads mapped in the Control cohort by ~ 77%, and ~ 67% in Fosfomycin cohort (Fig. 2). Little change occurred in mapping rates for the Ampicillin and Ciprofloxacin Cohorts.

### A large portion of uncharacterized scaffolds are not present in antibiotic treated mice gut microbiomes

**Fig. 3:**
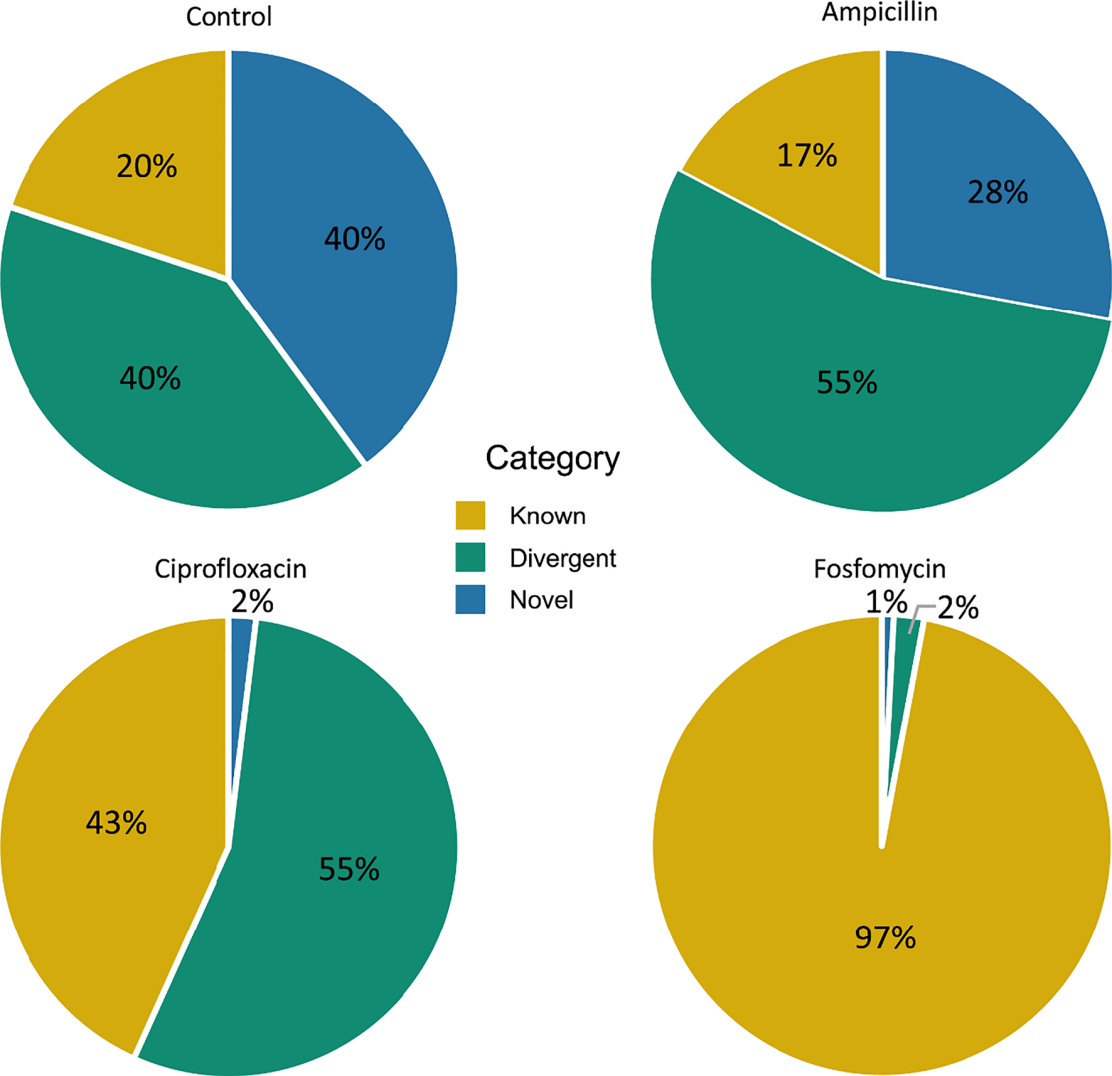
Composition of different categories of genomic scaffolds in each cohort. For each cohort, the assembled scaffolds are classified into one of the three categories: known, novel, and divergent. The classification is based on the degree of similarity between the predicted genes on scaffolds and known genes in the NCBI database. Novel scaffolds have BLASTn alignments that spanned less than 20% of bases and had an average gene identity below 60%. Known scaffolds had an average gene identity greater than 80%. Divergent scaffolds were all those that neither fit into novel or known (in Gene Prediction and Scaffold Divergence Classification section of Methods). The percentage of base pairs from scaffolds in each category relative to the total number of base pairs from all scaffolds is shown. Of note samples from the Fosfomycin cohort went through single-end sequencing while all other cohorts went through paired-end sequencing (see Methods and Discussion). The three categories of scaffolds (Novel, Known, and Divergent) were separately analyzed in downstream analysis

Through a characterization method described in Kowarsky et al.[22], we classified the assembled scaffolds into three categories: novel (BLASTn < 20% of nts and gene identity < 60%), known (BLASTn > 80% of nts), and divergent (neither novel or known; see Methods). We determined that 129,272 of the 313,519 Control scaffolds were collected from novel scaffolds likely to originate from genomes not fully characterized. Ampicillin-treated mice had 2,877 of their 12,438 scaffolds classified as novel. Interestingly, the Ciprofloxacin group only had 274 of its 25,171 scaffolds classified as novel. Lastly, Fosfomycin-treated group had 192 of its 5,331 scaffolds classified as novel. Because different scaffolds had different lengths, we further analyzed the portion of base pairs assigned to each category to normalize the effect of scaffold length. Control had 42% of scaffold base pairs assigned to the novel characterization (Fig. 3). Ampicillin, Ciprofloxacin, and Fosfomycin had 28%, 2%, and 1% of scaffold base pairs assigned to novel, respectively. In addition, Control had ~ 40% of their predicted genes come from novel scaffolds (Additional file 1: Table 1). Ampicillin, Ciprofloxacin, and Fosfomycin treatment groups had 24%, 2%, and 2% of their predicted genes come from novel scaffolds, respectively (Table 1). Markedly, antibiotic treated mice had a large reduction in scaffolds, predicted genes, and reads coming from novels scaffolds (Fig. 3, Additional file 1: Table 1). Further break down of the characterization of scaffolds for each cohort can be found in Additional file 1: Table 1.

**Table 1:**
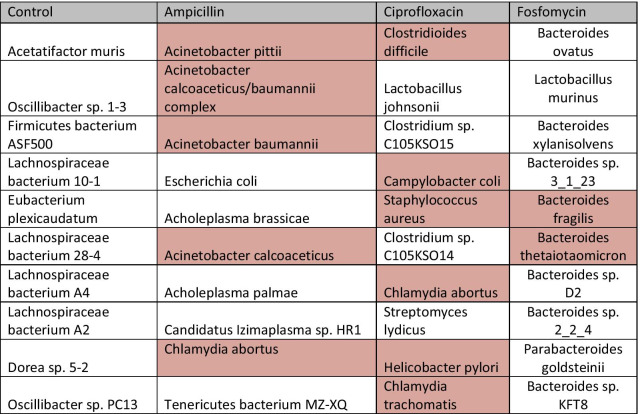
Top species by the number of contigs for the control and antibiotic treatment cohorts

For each cohort (either control or antibiotic treated groups), the top 10 species that have the highest number of assembled contigs are shown. Red shading indicates opportunistic pathogens. All three antibiotic treated cohorts, but not the control cohort, have opportunistic pathogens among the top 10 species

### Antibiotic treatment selects for opportunistic pathogens

**Fig. 4:**
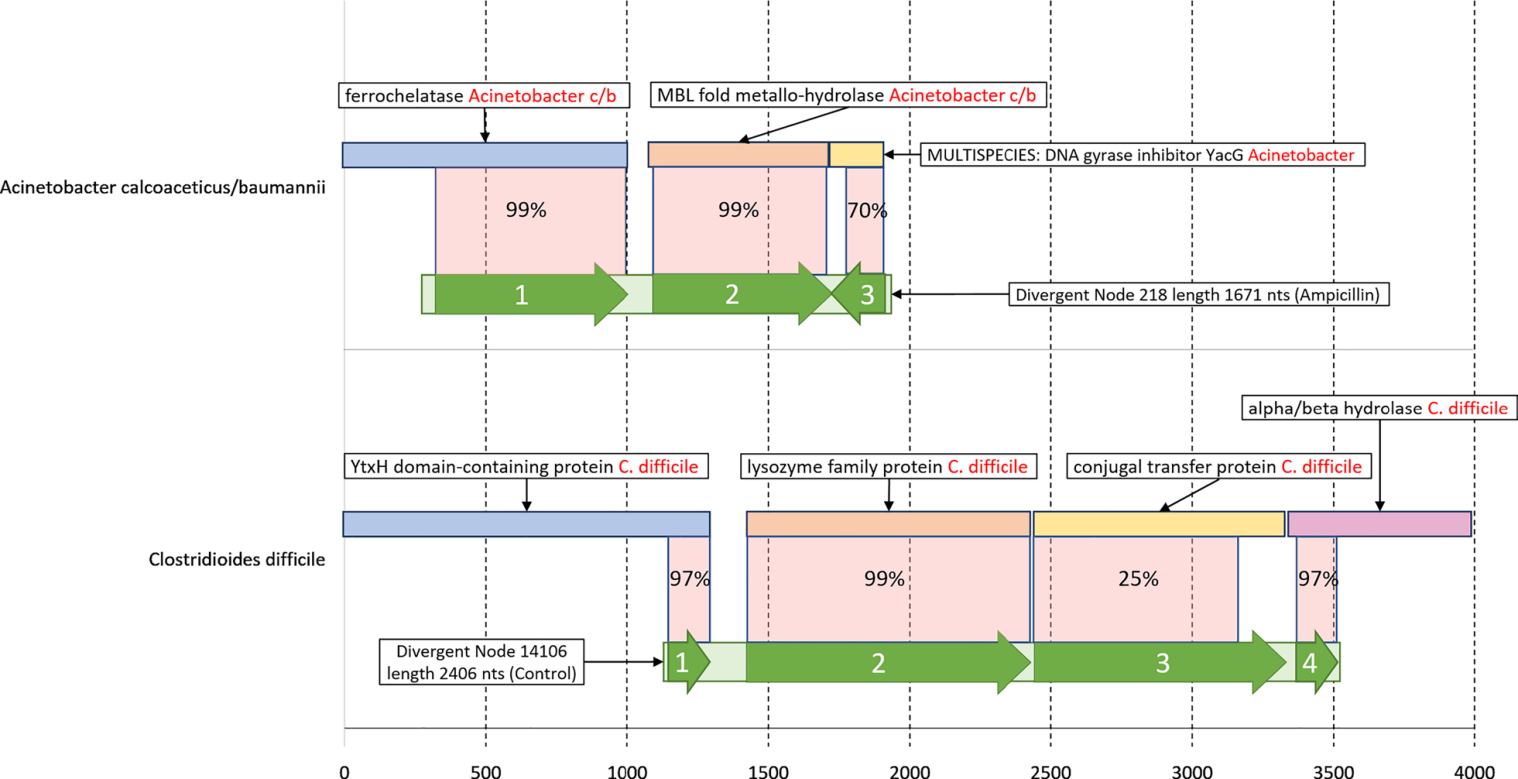
Gene track visual of two scaffolds that are likely from uncharacterized pathogen genomes (or epichromosomal elements). Labelled percentages are percent identity of query (predicted genes on the scaffolds) to subject (target proteins in the database). Transparent red boxes represent query coverage to subject. Top: an *Acinetobacter calcoaceticus/baumannii* scaffold (node 218) from the Ampicillin-treated cohort with a length of 1671 nucleotides (nts), with 3 predicted genes (green track) mapped to their targets. Bottom: a *Clostridioides difficile* scaffold (node 14,106) from the control cohort with a length of 2406 nts with 4 predicted genes (green track)

Higher level analysis was performed through taxonomic profiling based on a lowest common ancestor approach briefly described in methods. Lowest common ancestor assignment revealed that most control cohort scaffolds only had enough information to be assigned to the phylum level, with Firmicutes being the top phylum. Some control scaffolds were able to be identified at the species level and the top 10 species were from the Firmicutes phylum and Clostridiales order with the initiation of divergent branching occurring at the family level (Table 1). A total of 1,878 unique bacterial species with at least 5 scaffolds each were found in the control cohort. Conversely, Ampicillin, Ciprofloxacin, and Fosfomycin-treated groups only had 44, 108, and 49 unique bacterial species assigned with at least 5 scaffolds, respectively. Unlike the control cohort, the top 10 species present in each antibiotic treatment cohort had opportunistic pathogens present. Most notably, Ampicillin treated animals presented with *Acinetobacter calcoaceticus/baumannii* complex and *Chlamydia abortus* species of opportunistic pathogens (Table 1, highlighted in red). The top 10 species for Ciprofloxacin treated animals included opportunistic pathogens *Chlamydia abortus*, *Clostridioides difficile*, and *Staphylococcus aureus* (Table 1, highlighted in red). Fosfomycin’s top 10 species included *Bacteroides fragilis* and *Bacteroides thetaiotaomicron*, common gut microbes known for pathogenesis outside of the gut system (Table 1, highlighted in red). Importantly, some scaffolds we identified are likely from uncharacterized genomes (or epichromosomal elements) of specific pathogen species, as they harbor genes that are similar to, but distinct from, known genes of pathogen origin (Fig. 4).

### Quantitative analysis of read counts reveals opportunistic pathogen selection following antibiotic treatment

**Fig. 5:**
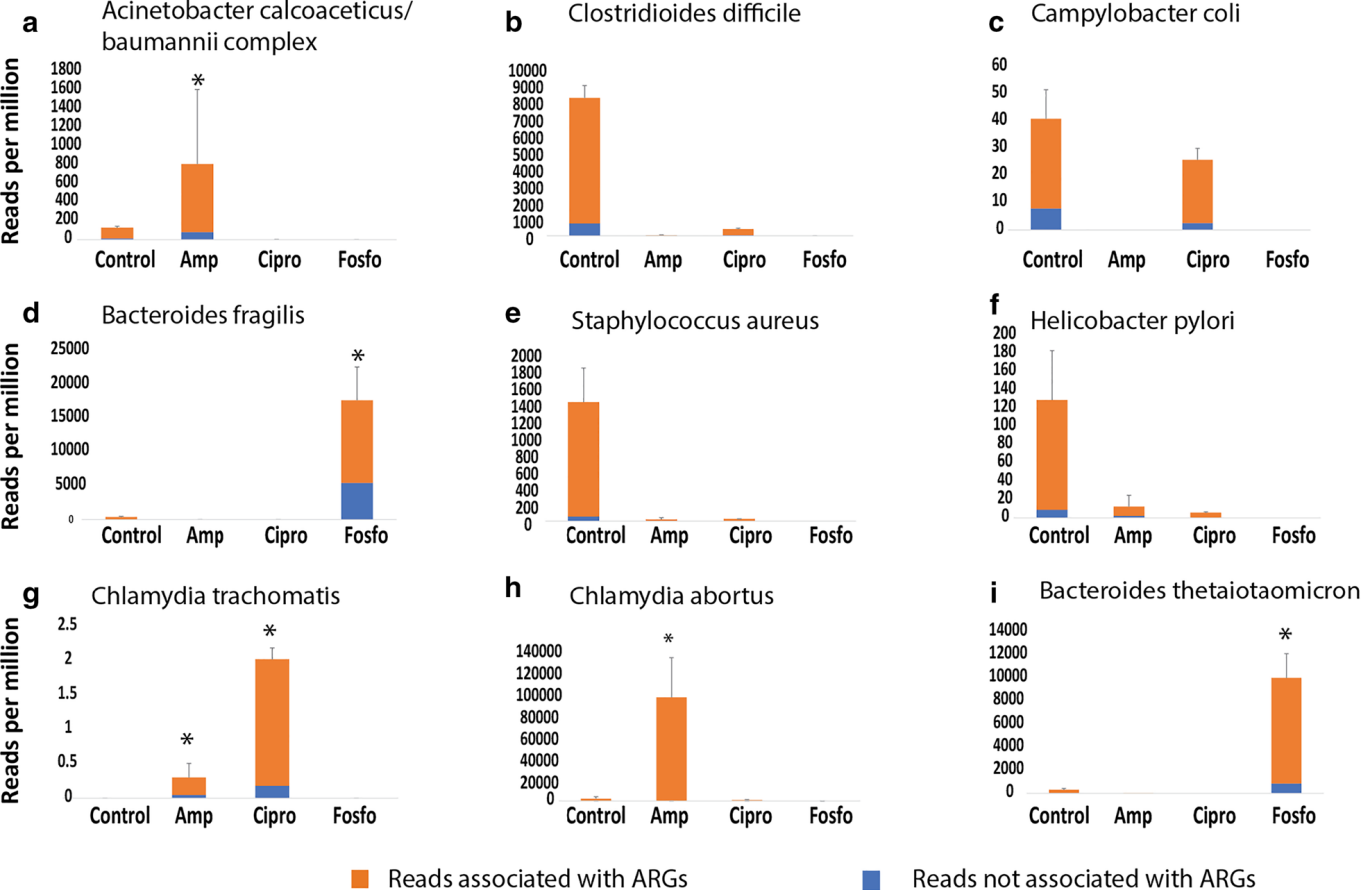
Opportunistic pathogen and antibiotic resistant gene abundance. The height of each bar in each panel indicates the normalized read counts (reads per million) for the opportunistic pathogen in a specific antibiotic-treated cohort. The blue and orange portion of the bar indicates the normalized read counts associated with ARGs (antibiotic resistant genes) and non-ARGs, respectively. Reads associated with ARGs (blue portion) were obtained by mapping the predicted genes to CARD (Comprehensive Antibiotic Resistance Database). *: Benjamini-Hochberg (BH) adjusted *p *value < 0.05

Employing a quantitative analysis (see Methods) based on reads mapped to predicted genes harbored by opportunistic pathogens, we determined that *Chlamydia abortus* and *Acinetobacter calcoaceticus/baumannii* complex had roughly 40-fold and 7-fold more normalized read counts in the Ampicillin group than in the Control group, respectively (Fig. 5). *Chlamydia trachomatis,* which was undetectable in Control group, registered a modest increase in normalized read counts in Ampicillin and Ciprofloxacin treated mice. The Fosfomycin cohort had > 30-fold more normalized reads mapped to both *Bacteroides fragilis* and *Bacteroides thetaiotaomicron* than the Control cohort. According to our analysis the opportunistic pathogen with the highest relative abundance (read counts relative to/normalized by the total microbiome reads from each sample) following antibiotic treatment was *Bacteroides fragilis*, which had on average 17,678 reads per million in the Fosfomycin-treated samples. This is a 46-fold increase in the relative abundance from the control group, where this opportunistic pathogen had on average 376 reads per million (Fig. 5).

### Higher number of antibiotic resistant genes in antibiotic selected opportunistic pathogen populations

**Fig. 6:**
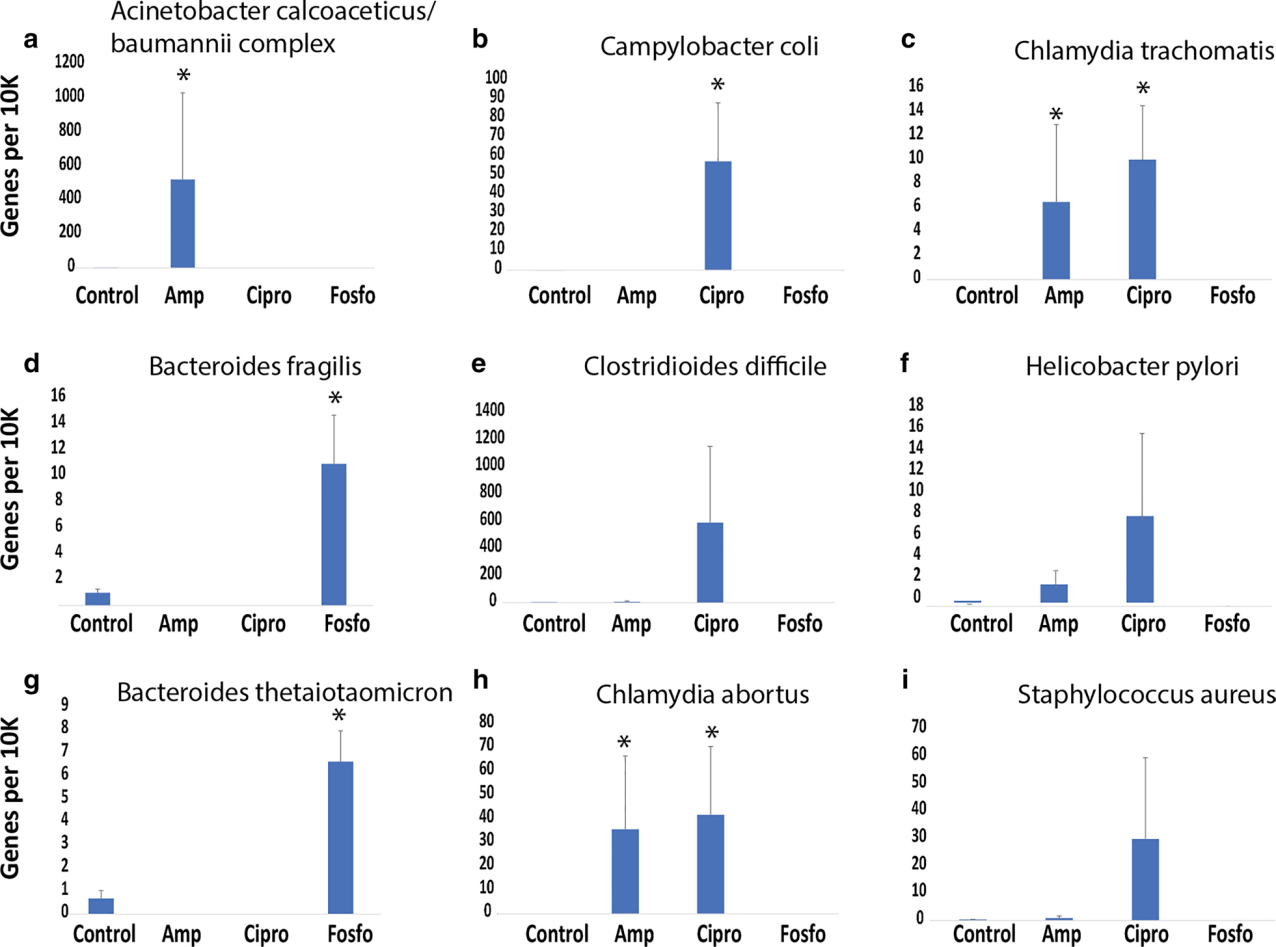
Opportunistic pathogen’s antibiotic resistance diversity profile. Y axis: number of genes associated with antibiotic resistance (normalized as per 10 K predicted genes). X axis: the control cohort and the three antibiotic-treated cohorts. *: Benjamini-Hochberg (BH) adjusted *p *value < 0.05

Some top opportunistic pathogen species selected by antibiotics (Table 1) do not show an increase in relative abundance after antibiotic treatment compared to control (Fig. 5). To examine other changes of these opportunistic pathogen populations in antibiotic treated cohorts, we assessed the Antibiotic Resistant Gene (ARG) profile. We determined that the *Acinetobacter calcoaceticus/baumannii* subpopulations selected for in Ampicillin treated mice harbored a greater number of antibiotic resistant genes (328 ARGs) than the entire *Acinetobacter calcoaceticus/baumannii* population in the Control cohort (13 ARGs). The difference in antibiotic resistant gene diversity is even more stark when comparing these numbers relative to the total number of predicted genes in their respective sample. For example, the average number of relative *Acinetobacter calcoaceticus/baumannii* antibiotic resistant gene counts was 520 (normalized as per 10 K gene) in Ampicillin treated mice and 0.17 in the Control cohort (Benjamini-Hochberg (BH) adj. *p *value = 0.00075) (Fig. 6). Additionally, antibiotic resistant gene counts in C*hlamydia abortus* (BH adj. *p *value = 0.0001) and *Chlamydia trachomatis* (BH adj. *p *value = 0.00075) exhibited a statistically significant increase in Ampicillin cohort compared to Controls. We further examined the diversity profile of each treatment group and found that Ciprofloxacin treated mice had well over 500-fold more antibiotic resistant genes associated with *Campylobacter coli* (BH adj. *p *value = 0.0037) than Control (57 ARGs vs 0.09 ARGs, Fig. 6). Similar to Ampicillin cohort, we observed a statistically significant uptick in the number of antibiotic resistant genes associated with both species of C*hlamydia* in Ciprofloxacin treated mice*.* Lastly, Fosfomycin treated mice had an average of 10.8 and 6.2 antibiotic resistant genes in *Bacteroides fragilis* and *Bacteroides thetaiotaomicron*, respectively. For both species of *Bacteroides*, the number of relative antibiotic resistant gene counts in Fosfomycin treated mice is > fivefold more than that in the control.

## Discussion

Urinary tract infection (UTI) is the leading cause of hospital acquired infections worldwide [26]. Previous studies suggest UTIs make up roughly 40% of all nosocomial infections in the USA [27, 28]. However, recent guidelines for prevention of catheter-associated UTIs and implementation of evidence-based interventions have resulted in significant decreases in nosocomial UTIs [29]. Despite the improvements in best practices and nosocomial UTI rates, the Centers for Disease Control (CDC) reports that UTIs are still among the most common type of hospital-associated infection in the USA (https://www.cdc.gov/hai/ca_uti/uti.html). Oral antibiotic administration to treat nosocomial infections, like hospital acquired UTIs, have been implicated in gastrointestinal microbiota dysbiosis, and secondary infections in human and mouse studies [30–34]. Furthermore, these treatments often select for an antibiotic resistant population, likely from an antibiotic resistant (AR) reservoir [3].

Our results strongly support the previously described hypothesis with direct evidence of gut microbiota dysbiosis, opportunistic pathogen selection, and a higher percentage of selected opportunistic pathogens harboring antibiotic resistant genes occurring after oral antibiotic administration. Here, the reduction of our total sequencing reads, characterized scaffolds, and number of unique species present define the state of dysbiosis after antibiotic administration. Furthermore, the shift from Firmicutes to Proteobacteria, that we present in our results, is a known state of dysbiosis after antibiotic treatment [35] and during disease [36–38]. Importantly, our scaffold assembly and lowest common ancestor assignment approach allowed us to identify which top species were selected for after antibiotic treatment. The assembly of short reads into longer scaffolds before aligning to reference sequences decreased the number of ambiguous alignments and increased the number of reads that can be mapped to reference databases. The lowest common ancestor approach increased taxonomic resolution for longer scaffolds with multiple predicted genes. These methods can be applied to other metagenomic studies to potentially gather more information from the complex metagenome datasets compared to traditional read-mapping based methods. Many studies have correlated nosocomial infection of *Acinetobacter calcoaceticus/baumannii* complex after antibiotic administration for a primary nosocomial infection [4, 39]. We provide direct evidence for an increase of relative abundance of *Acinetobacter calcoaceticus/baumannii*, *Chlamydia abortus*, *Chlamydia trachomatis*, *Bacteroides fragilis*, and *Bacteroides thetaiotaomicron* after various antibiotic treatment. For other pathogens studied, for example *Campylobacter coli* in the Ciprofloxacin cohort, we found that while the relative abundance is not increased, the numbers of ARGs are, suggesting more ARG-enriched subpopulations were selected by the treatment. Interestingly, our previous study suggested that ARGs enriched in cohorts treated by one class of antibiotics are often resistant to other classes of antibiotics [14]. We also observed that some opportunistic pathogens have more ARG-associated reads in control compared to antibody-treated groups (Fig. 5), probably because the relative abundance of these pathogens was reduced so much after antibiotic treatment that any reads coming from them, including ARG-associated reads, became almost undetectable.

We acknowledge several limitations of our studies. First, the number of animals in each cohort is small, and some animals were not included in the analysis due to insufficient number of sequencing reads. It is unknown whether the excluded animals would have any impact on the final results. Second, paired-end sequencing was completed on all samples except the entire Fosfomycin cohort, which was single-end sequenced. This potentially had several effects on our Fosfomycin results. For example, the number of scaffolds generated that passed our quality control measures were far lower in Fosfomycin than other cohorts. This was likely due to the method of assembly differences necessary to build the scaffolds, as reported in our methods. Additionally, the assembly process may have contributed to the differences seen in the characterization of scaffolds, where a larger portion of control and Fosfomycin scaffolds were characterized as novel. This large portion of novel scaffolds is expected for the control cohort as there is substantial evidence for a large unknown population of microbiota present in unperturbed gut microbiome [40]. However, the assembly pipeline we used, metaSPAdes, is optimized to use pair-end reads and there is a possibility the assembly process of metagenomic single-end reads into scaffolds won’t maintain the high confidence found in the pair-end assembly protocol [18]. The decision to sequence Fosfomycin cohorts single-ended was made in the beginning of this project as it was not clear if paired-end sequencing would generate enough reads for a good coverage of the gut microbiome. As subsequent experiments revealed paired-end sequencing not only generated enough reads but also provided higher quality reads for assembly, we switched to paired-end sequencing for all other samples. However, we feel it is still important to report the findings from the single-end-sequenced Fosfomycin cohorts as the observed pattens (selection for opportunistic pathogens and enrichment of ARGs) are consistent with other cohorts with slightly different (paired-end sequencing) techniques. A third limitation in this pipeline is the difficulty in differentiating divergent contigs from plasmids and free phages which may have been present. This limitation may be mitigated with further downstream analysis to include a plasmid and phage identifier tool. A final limitation is the use of a mouse model as there are many differences at the cellular level as well as the gross anatomy level from a mouse to a human. However, overall the gut microbiota of mice and humans is mostly comprised of Bacteroidetes and Firmicutes [41]. Furthermore, the amount of knowledge on mouse gastroenterology, genetics, and microbiome composition provide ample advantage on any other model for microbiota research [42]. Therefore, we believe the findings from our mouse UTI model potentially revealed a general mechanism where the use of single oral antibiotic treatment directly induces the expansion of opportunistic pathogens and ARG-enriched subpopulations in the gut microbiome, which may subsequently lead to secondary nosocomial infections. While more work, especially controlled clinical studies, are warranted to confirm this mechanism in humans, this raised a possibility that we may need to consider combination antibiotic treatments to suppress the expansion of opportunistic pathogens during an antimicrobial therapy. New studies are being conducted to investigate this possibility.

## Supplementary Information

**Additional file 1: Table 1**. Descriptive statistics of data processed by the analysis pipeline. For each sample in each cohort, the number of basepairs (BPs) in assembled contigs, the number of reads mapped to scaffolds, the number of predicted genes on scaffolds, and number of scaffolds classified into each of the three categories (novel, known, divergent) are given.

## Data Availability

All of the raw sequencing reads were deposited in Sequence Read Archive (sra@ncbi.nlm.nih.gov) with an accession number SRP152866.
